# Cerebral small vessel disease lesion segmentation methods: A systematic review

**DOI:** 10.1016/j.cccb.2025.100396

**Published:** 2025-09-19

**Authors:** Jolene Phelps, Manpreet Singh, Cheryl R. McCreary, Caroline Dallaire-Théroux, Ryan G. Stein, Zacharie Potvin-Jutras, Dylan X. Guan, Jeng-liang D. Wu, Amelie Metz, Eric E. Smith

**Affiliations:** a Department of Clinical Neurosciences, University of Calgary, Calgary, Canada; b Department of Mechanical Engineering, University of Victoria, Victoria, Canada; c Division of Medical Sciences, University of Victoria, Victoria, Canada; d Multiomics Investigation of Neurodegenerative Diseases (MIND) Laboratory, Montréal, Canada; e Département de pharmacologie et physiologie, Faculté de médecine, Université de Montréal, Montréal, Canada; f Institut de génie biomédical, Université de Montréal, Montréal, Canada; g Centre de Recherche de l’Institut Universitaire de Gériatrie de Montréal (CRIUGM), Montréal, Canada; h Department of Radiology, University of Calgary, Calgary, Canada; i Hotchkiss Brain Institute, University of Calgary, Calgary, Canada; j Department of Clinical Neurosciences, Hôpital de l'Enfant-Jésus, CHU de Québec, Université Laval, Québec, QC, Canada; k Centre de Recherche du CHU de Québec, Centre hospitalier de l'Université Laval (CHUL), Université Laval, Québec, QC, Canada; l Aging, Mobility, and Cognitive Health Laboratory, University of British Columbia, Vancouver, BC, Canada; m Djavad Mowafaghian Centre for Brain Health, University of British Columbia, Vancouver, BC, Canada; n Centre for Aging SMART at Vancouver Coastal Health, Vancouver Coastal Health Research Institute, Vancouver, BC, Canada; o Department of Physical Therapy, Faculty of Medicine, University of British Columbia, Vancouver, British Columbia, Canada; p Department of Physics, Concordia University, Montréal, Québec, Canada; q School of Health, Concordia University, Montréal, Québec, Canada; r Centre ÉPIC, Montreal Heart Institute, Montréal, Québec, Canada; s Vulnerable Brain Lab, Department of Anatomy and Cell Biology, Schulich School of Medicine and Dentistry, Western University, London, Ontario, Canada; t Douglas Research Centre, Montreal, Quebec, Canada; u Department of Psychiatry, McGill University, Montreal, Quebec, Canada; v Integrated Program in Neuroscience, McGill University, Montreal, Quebec, Canada

**Keywords:** Magnetic resonance imaging, Leukoaraiosis, Cerebral infarcts, Microbleeds, Perivascular spaces

## Abstract

Cerebral small vessel disease (CSVD) can manifest as brain lesions visible on magnetic resonance imaging, including white matter hyperintensities (WMH), cerebral microbleeds (CMB), perivascular spaces (PVS), lacunes, and recent small subcortical infarcts (RSSI). Detection and segmentation of these imaging markers can provide valuable information on brain health, including prevention and treatment of dementia. However, manual segmentation is cumbersome, especially for large cohorts in research studies. There has been extensive research into the development of automated tools using machine learning to increase accuracy and efficiency in lesion segmentation. This systematic review aimed to summarize novel automated methods developed over the last 10 years that segment CSVD lesion types and have been validated on a population with or at risk for CSVD (*e.g.,* older adults, those with cognitive disorders, or those with vascular risk factors). A search on Web of Science and PubMed yielded 2764 studies, of which 89 were included after screening and full text review. 59 of these methods segmented WMH, 23 detected or classified CMB, 6 detected or segmented PVS, 5 detected, classified, or segmented lacunes, and 2 segmented RSSI. Of these, 30 studies (23 for WMH, 5 for CMB, 1 for PVS, and 1 for lacunes) included links to download code or pre-trained models, including one commercial tool, and one that relied on a commercial tool for input. Overall, this review found good evidence for high quality tools available for WMH segmentation, with fewer tools available to accurately segment other CSVD lesion types.

## Highlights

• Systematic review of segmentation methods for cerebral small vessel disease lesions.
• Good evidence for validated methods for segmenting white matter hyperintensity.
• Fewer methods for microbleeds, perivascular spaces, and lacunes.

**GLOSSARY**

CAA: cerebral amyloid angiopathy; CADASIL: cerebral autosomal dominant arteriopathy with subcortical infarcts and leukoencephalopathy; CMB: cerebral microbleed; CNN: convolutional neural network; CSVD, cerebral small vessel disease; DER: detection error rate; DSC: DICE similarity coefficient; FP: false positive(s); FP_avg_: average number of false positives per scan/subject; FNR: false negative ratio; FPR: false positive ratio; ICH: intracerebral hemorrhage; k-NN: k-nearest neighbor; MR: magnetic resonance; MRI: magnetic resonance imaging; OER: outline error rate; PPV: positive predictive value; PVS: perivascular spaces; SVM: support vector machine; T2DM: type 2 diabetes mellitus; TIA: transient ischemic attack; TPR: true positive ratio (also referred to as recall or sensitivity); WM: white matter; WMH, white matter hyperintensity

Alt-text: Unlabelled box

## 1.0 Background

**Fig. 1:**
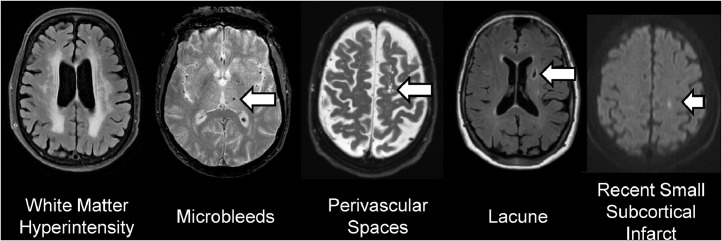
Lesions caused by cerebral small vessel disease (arrows).

Vascular disease and dysfunction can cause strokes and other forms of damage to the brain and is the second most common cause of dementia. Cerebral small vessel disease (CSVD), defined as diseases of the small arteries, capillaries and veins, is particularly common with aging and underlies much of the vascular risk for dementia. These diseases can cause types of brain injury visible on magnetic resonance imaging (MRI), including white matter hyperintensities (WMH) of presumed vascular origin, perivascular spaces (PVS), cerebral microbleeds (CMB), lacunes of presumed vascular origin, and recent small subcortical infarcts (RSSI) [25] (Fig. 1). To facilitate research on cases, prevention, and treatment of dementia, there has been intense interest in identifying and segmenting these lesions on MRI.

The rapid evolution of machine learning offers an unparalleled opportunity for more efficient, accurate identification and segmentation of radiological lesions. Among the lesions caused by CSVD, WMH has so far received the most attention. However, there are also important challenges to accurate segmentation, whether supervised (*i.e.,* requiring manual segmentations as a reference during training), unsupervised (*i.e.,* without ground truth labels, *e.g.,* using clustering methods in combination with anatomical knowledge to identify patterns), weakly supervised (*i.e.,* trained with incomplete or inexact labels), or rule-based (*i.e.,* with pre-defined rules or thresholds based on image characteristics). CSVD lesion types such as WMH have ill-defined boundaries, loads vary substantially across patients, co-occurring pathologies can confound assessments, and contrast and image quality can vary substantially across different scanners and sites. Yet, there is a clinical need to enable multicenter studies and clinical trials, leading ultimately to risk for false positives and negatives. Additionally, signal intensities and spatial distributions vary depending on the specific disease or neurological condition [7], meaning that methods developed in one context (*e.g.,* to identify white matter lesions in patients with multiple sclerosis) require validation when applied to other contexts, such as WMH of presumed vascular origin.

Given these challenges, it is important to understand which segmentation tools are valid for CSVD segmentation and to what extent. This need is growing given that MRI imaging is increasingly being incorporated into epidemiological studies and clinical trials, and that large MRI databases, such as the UK Biobank and Alzheimer’s Disease Neuroimaging Initiative (ADNI), are available for access for secondary analyses. While some investigators will want to derive and validate in-house segmentation methods specific to their own dataset, others are looking for “off-the-shelf” solutions. Previous systematic reviews have focused mostly on advances in methods, with validity and accessibility to the research community often being secondary considerations. Thus, in our view there is a need for a systematic review of segmentation methods for CSVD with a primary emphasis on the validity of the tools, their derivation, and validation in an appropriate population, and their availability to the wider research community. The objective of this review was to identify segmentation tools for WMH, PVS, CMB, lacunes and RSSI, summarizing their validity and availability to the research community for download and use.

## 2.0 Methods

The review was conducted according to the Preferred Reporting Items for Systematic Reviews and Meta-Analyses (PRISMA) [72]. All data used in this study are contained in this article and the accompanying Appendix B.

### 2.1 Literature search

The protocol for this systematic review was published on Open Science Framework (DOI 10.17605/OSF.IO/K7RQF) on January 30, 2024, and followed methods from the Joanna Briggs Institute Manual for Evidence Synthesis. Two databases were searched: MEDLINE/PubMED and Web of Science on December 21, 2023, and again on September 19, 2024. The search terms were extracted from identified key articles and in consultation with respective MeSH headings [7,25,65]. The detailed search strategy for each database can be found in Appendix A.

### 2.2 Inclusion and exclusion criteria

Inclusion criteria for the search were:

1. Study includes presentation and validation of a method for segmenting one or more of the following lesions of presumed vascular origin from human brain magnetic resonance (MR) images in persons with aging, cognitive disorders, or cerebrovascular diseases: WMH, lacunes, CMB, PVS, cortical superficial siderosis, cortical cerebral microinfarcts, RSSI.
2. Segmentation accuracy against a reference standard was evaluated.
3. Enough information was provided to replicate or apply the segmentation method.
4. The study was on humans (*i.e.,* animal studies were excluded).
5. The study was published in English in a peer-reviewed journal on or after December 20, 2013 (10 years from the first search).

We excluded studies where:

1. The focus was on pre-processing of MR studies.
2. The output was classification of patients (*e.g.,* to predict which patients harbored a lesion) instead of segmenting the lesion.
3. The study population was humans but *ex vivo* imaging was done.

### 2.3 Eligibility screening

A team of trained reviewers independently reviewed and screened titles and abstracts using Covidence® software with a minimum of two reviewers per title and abstract. Full text review was subsequently conducted independently, with a minimum of two reviewers per text. All conflicts were resolved through consensus review.

### 2.4 Data extraction

Data extraction was completed by an individual reviewer and checked at minimum by a second reviewer. Extracted information included the methods employed for segmentation, MRI scanners used, validity of segmentation compared with a reference standard (usually manual segmentation), requirements for pre-processing (yes/no), segmentation tool dependencies, time to set up and process scans, and availability for use by other researchers. We specifically sought information on sensitivity (TPR), specificity, accuracy, dice similarity coefficient (DSC), false negative ratio (FNR), false positive ratio (FPR), F1 score, precision, recall, average false positives per subject, 95th percentile Hausdorff distance (HD95), average volume distance (AVD), and other specified performance metrics of the proposed segmentation method compared to the reference standard.

### 2.5 Assessment of study quality

Methodological quality was assessed using the METhodological RadiomICs Score (METRICS), a scoring tool developed to assess methodological quality of radiomics research [47]. METRICS was developed using a modified Delphi protocol with a large international panel of 59 panelists from 19 countries to select and rank items and categories, resulting in 30 items within 9 categories, listed in descending order of importance: study design, imaging data, image processing and feature extraction, metrics and comparison, testing, feature processing, preparation for modeling, segmentation, and open science. Scoring was conducted by two individual reviewers, with consensus review for scores that did not match the same category (*e.g.*, excellent *vs* good).

## 3.0 Results

**Fig. 2:**
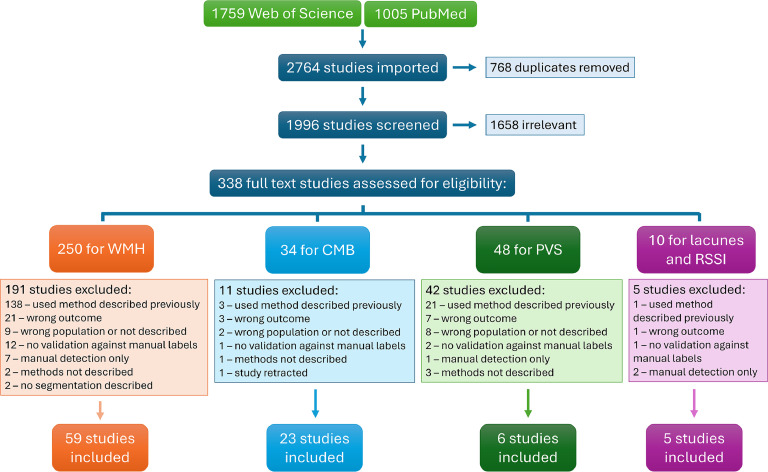
PRISMA flow diagram of the systematic literature search for studies presenting novel methods to segment white matter hyperintensities (WMH), cerebral microbleeds (CMB), perivascular spaces (PVS), lacunes, and recent small subcortical infarcts (RSSI), validated on a population with or at risk for cerebral small vessel disease.

A total of 1996 papers were found in the search after removal of 768 duplicates. After screening, a total of 338 studies were reviewed in full text after which 249 were excluded, leaving 89 papers for review (Fig. 2). The most common reason for exclusion at the full text stage was that the paper described use of a previously published tool in applied research. Of the included papers, 59 segmented WMH, 23 detected and classified CMB, 6 segmented PVS, 5 detected, classified, or segmented lacunes, and 2 segmented RSSI in addition to lacunes (note: papers that segment more than one lesion type are counted in multiple categories). No publications were found that described segmentation of cortical superficial siderosis or cortical cerebral microinfarcts. The complete extraction table can be found in Appendix B.

### 3.1 Quality review

Methodological quality, assessed using METRICS [47], was excellent (scoring 80–100) in 35/89 (39 %), good (scoring 60–80) in 52/89 (58 %), and moderate (scoring 40–60) in 2/89 (3 %) (Table 1). Studies describing detection/segmentation of PVS and lacunes were generally of worse quality: only 1/6 scored excellent for PVS and 1/5 scored excellent for lacunes. Due to the selection criteria for the review, all included studies were required to have a high-quality reference standard, clear description of the segmentation methodology, formal evaluation, transparent reporting, and a calibration assessment.

**Table 1:** Number of studies meeting quality standard or characteristics outlined in METRICS.

Metric		Total (*N* = 89)	WMH (*N* = 59)	CMB (*N* = 23)	PVS (*N* = 6)	Lacune (*N* = 5)	RSSI (*N* = 2)
Overall score		Number of studies					
	Excellent	35	27	8	1	1	1
	Good	52	32	13	5	4	1
	Moderate	2	0	2	0	0	0
	Poor	0	0	0	0	0	0
Selected characteristics		Number of studies					
	Multi-centre	49	38	9	3	1	1
	Appropriate image preprocessing	84	56	21	6	5	2
	Appropriate dimensionality	68	48	16	3	5	2
	Uni-parametric imaging	42	17	21	4	2	1
	Comparison with simple or classical models	55	41	12	2	0	0
	Internal testing	81	52	21	6	4	1
	External testing	29	25	6	0	1	1
	Data availability	26	21	6	0	0	0
	Code availability	21	15	5	0	1	0
	Model availability	17	14	2	1	0	0

### 3.2 Methods with implementable code or models available

Of the included 89 studies, 30 (23 for WMH, five for CMB, one for PVS, and one for lacunes) included links to download implementable code or models as seen in Table 2. For WMH, five models are available as a toolbox, software, or pipeline, and two are available within FSL. Another 11 studies provide code for Python implementation, with five additionally providing pretrained models and four providing a Docker image. Two studies provided only a Docker image, and one study provides only a pretrained model. One model is only available commercially, while another requires results of another commercial product to function; the rest were not available for download or commercial products. For CMB, all five studies include links to publicly available source code with full python implementation of which three include pre-trained models. For PVS, one trained model is available [39], and for lacunes, the source code is available for one study [3].

**Table 2:** Studies providing code or implementable models for segmentation.

Reference	Lesion Type	Name of tool	Description	Link to download
[42]	WMH	W2MHS	Open-source MATLAB toolbox (requires SPM)	https://www.nitrc.org/projects/w2mhs
[34]	WMH	BIANCA	Available within FSL	https://fsl.fmrib.ox.ac.uk/fsl/docs/#/
[17]	WMH	CASCADE	*C*++ software (requires FSL)	https://github.com/Damangir/Cascade
[15]	WMH		Python implementation including pipeline, trained classifiers, and Docker image	https://nist.mni.mcgill.ca/white-matter-hyperintensities/
[44]	WMH	UBO Detector	Pipeline (requires MATLAB, SPM, and FSL)	https://www.cheba.unsw.edu.au/research-groups/neuroimaging/pipeline
[53]	WMH	sysu_media	Python implementation and Docker image	https://github.com/hongweilibran/wmh_ibbmTum
[43]	WMH	Dilated Saliency U-Net	Python implementation and Docker image for IAM	https://github.com/febrianrachmadi/lots-iam-gpu
[91]	WMH	LOCATE	Available within FSL/BIANCA	https://fsl.fmrib.ox.ac.uk/fsl/docs/#/
[105]	WMH	SC U-Net	Docker image	https://hub.docker.com/r/skipconnection/wmhchallenge_scunet/
[20]	WMH	jSTABL	PyTorch implementation	https://github.com/ReubenDo/jSTABL
[99]	WMH	StackGen-Net	Pretrained models and evaluation script for testing	https://github.com/spacl-ua/wmh-segmentation
[73]	WMH	pgs	Docker image	https://hub.docker.com/r/wmhchallenge/pgs
[113]	WMH	BAGAU-Net	Python implementation	https://github.com/Ericzhang1/BAGAU-Net
[92]	WMH	TrUE-Net	Python implementation, pretrained models, and Docker image	https://github.com/v-sundaresan/truenet https://hub.docker.com/repository/docker/wmhchallenge/fmrib-truenet_2
[55]	WMH	WHAT	Pipeline (requires FSL, PyTorch, and MATLAB)	https://www.nitrc.org/projects/what_v1
[95]	WMH	WHASA-3D	Commercial product from Qynapse	https://qynapse.com/qyscore/
[69]	WMH	HyperMapp3r	Pipeline (requires Python, ANTs, and Convert3D) and Docker image	https://hypermapp3r.readthedocs.io/en/latest/
[71]	WMH		MATLAB code, requires input results of trimmed mean outlier detection (commercial product)	https://github.com/kokhaur/WML-Segmentation-False-Positive-Detection
[93]	WMH	ConvNet	Pretrained PyTorch implementation	https://github.com/bthyreau/deep-T1-WMH
[115]	WMH	2D VB-Net	Python implementation, pretrained model, sample images with ground truth segmentations	https://github.com/simonsf/wmh-segmentation
[33]	WMH	CASCADE U	PyTorch implementation	https://github.com/gongt99/Cascade-U-Net-white-matter-hyperintensities-
[97]	WMH	SHIVA-WMH	Python implementation and pretrained models	https://github.com/pboutinaud/SHIVA_WMH
[75]	WMH	Instance loss functions	Python implementation for loss functions	https://github.com/BrainImageAnalysis/instance-loss.git
[21]	CMB		Python implementation, pretrained model, and subset of data	https://www.cse.cuhk.edu.hk/~qdou/cmb-3dcnn/cmb-3dcnn.html
[2]	CMB		Python implementation	https://github.com/Yonsei-MILab/Cerebral-Microbleeds-Detection
[76]	CMB	DEEPMIR	Python implementation	https://github.com/NAL-UTHSCSA/CMB_NHID_Segmentation
[90]	CMB	MicrobleedNet	Python implementation and pretrained models	https://github.com/v-sundaresan/microbleed-detection
[51]	CMB	TPE-Det	Python implementation and pretrained models	https://github.com/Yonsei-MILab/TPE-Det
[39]	PVS	mcPVS-Net	Pretrained model	https://github.com/huangnick/mcPVS-Net/tree/main
[3]	Lacunes	3D ResNet	Python implementation	https://github.com/Yonsei-MILab/Lacunes-Identification

### 3.3 White matter hyperintensity segmentation

A total of 59 studies met inclusion criteria and described novel methods for the segmentation of WMH of presumed vascular origin, detailed in Table 3. Most studies trained and tested their segmentation tools on local cohorts of participants. However, following the WMH Segmentation Challenge organized in association with MICCAI 2017 [48], many studies (23/42) published in 2018 or later utilized all or part of the available dataset from the challenge to train, test, or both train and test their models. The MICCAI training dataset includes 60 T1 and fluid attenuated inversion recovery (FLAIR) images from three scanners with manual annotation of WMH as binary masks, and the test dataset included 110 images from five different scanners, three of which were also used in the training dataset.

**Table 3:** Studies describing novel methods for segmenting white matter hyperintensities.

Ref	Seq / *Field strength*	Architecture (classification), *type*	Dataset (training/ testing)	Total scans (training/ validation/testing)	Performance metrics
[42] **	T1, FLAIR / *3T*	SVM compared to random forest, *S*	MCI, AD, or healthy controls	38 - leave-one-out CV	DSC: 0.67 for random forest, DSC: 0.54 for SVM
[108]	FLAIR / *1.5T*	Optimal threshold algorithm, *RB*	KLOSCAD - Range of WMH burdens	16 × 2 for derivation, 16 for testing	DSC: 0.76
[96]	T1, FLAIR, DWI / *1.5T*	Histographic characterization, *RB*	Acute ischemic stroke	20 for derivation, 10 for testing	DSC: 0.81, TPR: 0.76, specificity: 1.00
[101]	FLAIR / *3T*	Trimmed-likelihood estimator, EM algorithm, *RB+US*	Range of WMH burdens	82	DSC: 0.80, FNR: 0.20, FPR: 0.19
[102]	T1, FLAIR, T2 / *3T*	GMM-EVT, *US*	WM abnormalities	86	FLAIR only DSC: 0.80, multimodal DSC: 0.78
[88]	T1, FLAIR / *3T*	Bayesian Model Selection, EM algorithm, *US*	MRBrainS13 - T2DM and matched controls	19	DSC: 0.46, TPR: 0.38, AVD: 6.8
[81]	T1, FLAIR / *1.5T*	Random forest and MRF, *S*	ENVISion - Older with hypertension	18/6 – 4-fold CV	DSC: 0.61–0.76, TPR: 0.71–0.73, FP_avg_: 0.77–1.09 per slice
[34]**	T1, FLAIR / *3T*	k-NN, *S*	OPTIMA - MCI, AD, SCD; OXVASC - non-disabling stroke or TIA	21 for OPTIMA, 109 for OXVASC - leave-one-out CV	DSC: 0.52–0.75, FPR: 0.22–0.46, FNR: 0.26–0.45
[32]	T1, FLAIR / *1.5T*	Random forest with Adaboost, *S*	RUN DMC - CSVD on neuroimaging	312/50	TPR: 0.80, FP_avg_: 27–47 per volume
[17] **	T1, T2, FLAIR, PD / *1.5T*	Heuristic thresholding, SVM, *RB+S*	KHP-DCR - MCI, AD, or healthy controls	119	DSC: 0.85–0.91
[109]	T1, T2, FLAIR, PD / *1.5T*	SVM, region-scalable fitting, *RB+S*	ACCORD-MIND (multi-centre)	2/43	DSC: 0.81, TPR: 0.79, PPV: 0.84, TNR: 1.00
[79]	T1, FLAIR / *1.5T*	Hierarchical multi-threshold, SVM, *RB+S*	Brain infarct study and MCI study	28 total - leave-one-out CV	DSC: 0.74, accuracy: 0.89, USR: 0.27, OSR: 0.09
[16]	T1, T2*, FLAIR, T2/PD / *1.5–3T*	Linear regression, *S*	ADC - MCI, AD, and controls (multi-centre); PREVENT-AD - cognitively normal with parental AD / ADNI2/GO (multi-centre)	80 for ADC, 40 for PREVENT-AD – 10-fold CV External testing on 10 images from ADNI2/GO	DSC: 0.52–0.64, TPR: 0.52–0.71, PPV: 0.59–0.69, FPR: 0.0002–0.0014
[30]	T1, FLAIR / *1.5T*	2D CNN, *S*	RUN DMC - CSVD on neuroimaging	378/42	DSC: 0.79
[15] **	T1, FLAIR / *1.5–3T*	Random forest (highest performance compared to other classifiers), *S*	ADC - MCI, AD, and controls; NACC and ADNI2/GO - MCI, AD (all multi-centre)	70 for ADC, 32 for NACC, 46 for ADNI2/GO – 10-fold CV	DSC: 0.66–0.72, TPR: 0.62–0.79, PPV: 0.58–0.80, DER: 0.09–0.18, OER: 0.42–0.50
[68]	T1, FLAIR, T1-IR / *3T*	2D CNN, *S*	MRBrainS13 / healthy older subjects for UDES	20/96	DSC: 0.47, TPR: 0.82, DER: 0.16, OER: 0.83
[35]	T1, FLAIR / *1.5T*	2D CNN, *S*	Non-disabling lacunar or mild cortical ischemic stroke	167 total - 1-fold/1-fold	DSC: 0.69
[44]**	T1, FLAIR / *1.5–3T*	k-NN, *S*	SAMS - Older adults / OATS - Older adults (multi-centre)	10/40	DSC: 0.85, TPR: 0.91, specificity: 0.99, FPR: 0.03, accuracy: 0.99, DER: 0.04, OER: 0.22
[64]	T1, FLAIR / *3T*	Boosting based ensemble learning, *S*	AIBL - Range of WMH burdens	68/60	DSC: 0.79, TPR: ∼0.87, specificity: ∼0.99
[46]	FLAIR / *1.5–3T*	Voxel-wise logistic regression, *S*	MICCAI 2017 WMH Challenge* (multi-centre)	60/110	DSC: 0.70, TPR: 0.25, HD95: 17.03, AVD: 0.40, F1: 0.35
[53] **	T1, FLAIR / *1.5–3T*	2D U-Net, *S*	MICCAI 2017 WMH Challenge* (multi-centre)	60/110	DSC: 0.80, TPR: 0.84, HD95: 6.3, AVD: 0.22, F1: 0.76
[104]	FLAIR / *3T*	Multi-atlas-based detection and localization, *RB+US*	BIOCARD - Cognitively normal or MCI	15 for atlas generation, 120 for evaluation	DSC: 0.55–0.72
[82]	FLAIR / *1.5T*	2D CNN, *S*	Ischemic stroke / MRI-GENIE (multi-centre)	699/91/90 then validated on 144	*r* = 0.86, ICC: 0.84
[43] **	FLAIR / *3T*	2D Dilated Saliency U-Net using irregularity map, *S*	ADNI* (multi-centre)	10 × 3/10 × 3	DSC: 0.56, TPR: 0.47, PPV: 0.64
[91] **	T1, FLAIR / *3T*	Locally adaptive threshold estimation, *S*	OPTIMA - MCI, AD and controls; OXVASC - non-disabling stroke or TIA; MICCAI 2017 WMH Challenge* / validation on CADASIL cohort (multi-centre)	21 for OPTIMA, 18 for OXVASC, 60 for WMH challenge - leave-one-out CV external validation on 15 CADASIL	DSC: 0.64–0.79, TPR: 0.48–0.86
[105] **	T1, FLAIR / *3T*	2D U-Net with novel skip connection, *S*	MICCAI 2017 WMH Challenge* (multi-centre)	40/20	DSC: 0.78, TPR: 0.81, HD95: 7.36, AVD: 0.28, F1: 0.71
[6]	T1, T2, FLAIR / *1.5–3T*	CNN, *US*	AGES-Reykjavik - older adults; MICCAI 2017 WMH Challenge* (multi-centre)	30/25 for AGES, 60/110 for MICCAI	DSC: 0.62–0.77, TPR: 0.33–0.64, HD95: 10.97–24.49, AVD: 0.33–0.44, F1: 0.36–0.47
[58]	T1, FLAIR / *3T*	2D CNN with 2 U-shaped subnets, *S*	MICCAI 2017 WMH Challenge* (multi-centre) / ISLES challenge	52/8/15	DSC: 0.83, TPR: 1.00, PPV: 0.91, F1: 0.92, accuracy: 0.93, HD: 2.25
[19]	T1, FLAIR / *3T*	Logistic regression with NNR and Gaussian filter refinement, *S*	Normal Aging Study - Cognitively normal (multi-centre)	15/5	DSC: 0.78, TPR: 0.70, PPV: 0.85, FPR: 0.009
[77]	T1, FLAIR / *3T*	SVM and ResNet, *S*	MICCAI 2017 WMH Challenge* (multi-centre)	150 total – 10-fold CV	DSC: 0.92, TPR: 0.94, specificity: 0.91, accuracy: 0.92
[22]	T1, FLAIR / *1.5–3T*	2D U-Net, *S*	CNSR-III - Ischemic stroke or TIA, 1 or more CSVD sign on MRI / 2–4 CSVD markers on MRI (multi-centre)	824/30	DSC: 0.67, F1: 0.64
[59]	FLAIR, DWI / *1.5–3T*	2D U-Net with attention mechanism, *S*	ISLES challenge	26 cases total split into 518/224 images	DSC: 0.73, TPR: 0.94, HD: 2.63, accuracy: 0.94
[114]	T1, FLAIR / *3T*	2D U-Net with CRF, *S*	MICCAI 2017 WMH Challenge* (multi-centre)	60 total – 5-fold CV	DSC: 0.78, TPR: 0.77, HD95: 3.70, AVD: 0.10, F1: 0.67
[20] **	T1, FLAIR / *3T*	3D U-Net and HeMIS, *S*	MICCAI 2017 WMH Challenge*, MRBrainS18* (multi-centre)	60 for MICCAI, 7 for MRBrainS18 – 3-fold CV split 70/10/20 %	DSC: 0.59–0.78, HD95: 4.2–7.2
[56]	T1, FLAIR / *3T*	Anatomical knowledge-based 2D U-Net, *S*	MICCAI 2017 WMH Challenge* (multi-centre)	50/10	DSC: 0.83, TPR: 0.81, HD95: 4.16, F1: 0.79
[99] **	FLAIR / *3T*	3 orthogonal 3D CNNs, *S*	Extracranial carotid artery disease / ADNI3	20/20	DSC: 0.76, TPR: 0.67, HD95: 17.1, PPV: 0.84, F1: 0.73
[73] **	T1, FLAIR / *3T*	2D U-Net with multi-scale highlighting foregrounds, *S*	MICCAI 2017 WMH Challenge* (multi-centre)	40/20	DSC: 0.81, TPR: 0.82, HD95: 5.63, AVD: 0.19, F1: 0.79
[113] **	FLAIR / *3T*	Brain atlas guided attention U-Net, *S*	MICCAI 2017 WMH Challenge* (multi-centre), ABVIB*	48/6/6 for MICCAI, 24/3/3 for ABVIB	DSC: 0.80–0.82, TPR: 0.59–0.83, AVD: 0.14–0.16, F1: 0.60–0.78
[92] **	T1, FLAIR / *1.5–3T*	Triplanar ensemble U-Net, *S*	AD, MCI, or controls; OXVASC - non-disabling stroke or TIA; MICCAI 2017 WMH Challenge* (multi-centre)	Training: 9 for NDGEN, 18 for OXVASC, 60 for MICCAI; all validated on 110 from MICCAI	DSC: 0.89–0.95, TPR: 0.89–0.94, HD95: 1–1.2, AVD: 0.05–0.10, F1: 0.81–0.95
[110]	FLAIR / *1.5–3T*	2D U-Net, *S*	CSVD (multi-centre)	298 total - 60/20/20 %	DSC: 0.78, TPR: 0.85, PPV: 0.79
[40]	T1, FLAIR / *3T*	Half Gaussian mixture model, *US*	WHICAP - Range of WMH burdens	24	DSC: 0.87, TPR: 0.89, specificity: 0.99
[55] **	T1, FLAIR / *1.5–3T*	2D U-Net, SE-Net, and multi-scale features U-Net ensemble, *S*	MICCAI 2017 WMH Challenge*, CNSR / external validation on OATS - older adults (all multi-centre)	Internal: 60 total, 5-fold CV External 60/40	DSC: 0.62–0.83, TPR: 0.49–0.83, HD95: 5.2–9.9, AVD: 0.14–0.47, PPV: 0.75–0.92
[84]	FLAIR / *1.5–3T*	2D U-Net, *S*	CSVD (multi-centre)	870/160/126	DSC: 0.71, TPR: 0.65, PPV: 0.79
[13]	T1, FLAIR / *3T*	3D CNN and CRF, *S*	MICCAI 2017 WMH Challenge* (multi-centre)	36/12/12	DSC: 0.79, TPR: 0.67, HD95: 4.3, AVD: 0.15, F1: 0.70
[95] **	T1, FLAIR / *3T*	Non-linear diffusion and watershed parcellation, *RB+US*	ADNI - AD and controls; NIFD: FTD and controls; MEMORA: MCI, AD, unspecified	2/8 for ADNI, 2/13 for NIFD, 2/3 for MEMORA - combined scores	DSC: 0.79, TPR: 0.82, F1: 0.46, FPR: 0.22
[69] **	T1, FLAIR / *3T*	3D Bayesian U-Net, *S*	ONDRI - CVD, VCI, or PD; CAIN: nonsurgical carotid stenosis, LIPA: aphasia, VBH: CVD, VCI, or AD / MITNEC: severe WMH; CAIN: controls; ONDRI: CVD or PD; LIPA: controls or FTD; VBH: CVD or controls (multi-centre)	432/158	DSC: 0.89, TPR: 0.76, AVD: 0.10, F1: 0.75
[71] **	T1, FLAIR / *1.5T*	GLCM, random forest, local outlier factor algorithm, *US+S*	Cardiovascular risk factors	10/32 - 10-fold CV for training random forest	DSC: 0.30–0.89, TPR: 0.22–0.88, PPV: 0.48–0.90, FPR: 0.09–0.19
[93] **	T1, FLAIR / *1.5–3T*	3D Cross-domain U-Net, *S*	JPSC-AD (multi-centre)	4096/3598 (only 55 manually corrected)	DSC: 0.55
[115] **	T1, T2, FLAIR / *1.5–3T*	2D VB-NET, *S*	Range of WMH burdens from multiple datasets, including MICCAI 2017 WMH Challenge* (multi-centre)	5-fold CV on all datasets: multi-scanner sets: 849 and 20; independent sets: 102 and 74; MICCAI: 60	DSC: 0.78–0.79, TPR: 0.78–0.85, HD95: 4.9–10.3, AVD: 0.18–0.44, PPV: 0.72–0.81, F1: 0.64–0.76
[38]	T1, FLAIR / *3T*	LSLoss and V-Net, *SS*	HKU-SVD cohort, MICCAI 2017 WMH Challenge* (multi-centre)	HKU-SVD: 340/20 MICCAI: 60/110	DSC: 0.75–0.83, TPR: 0.61–0.90, HD95: 3.33–6.04, AVD: 0.11–0.31, F1: 0.72–0.92
[33] **	FLAIR / *3T*	2D U-Net with combined loss function, *S*	CAMERA	176/18/77	DSC: 0.61, TPR: 0.62, PPV: 0.64
[23]	FLAIR / *3T*	2.5D U-Net - compares VGG16, VGG19, ResNet152, EfficientNetB0 feature extractors, *S*	Control and vascular datasets / MICCAI 2017 WMH Challenge* (multi-centre)	40/20/60 – 5-fold CV for training/validation	VGG16 and VGG19 had similar or superior performance based on F measure
[52]	FLAIR / *1.5–3T*	2D U-Net with bottleneck attention modules, *S*	In house datasets with WMHs present / MICCAI 2017 WMH Challenge* (multi-centre)	239/170	DSC: 0.72, TPR: 0.48, HD95: 9.28, PPV: 0.83, F1: 0.59
[97] **	T1, FLAIR / *3T*	3D U-Net, *S*	MICCAI 2017 WMH Challenge* and MRi-Share* / UK Biobank, MRi-Share, MICCAI (multi-centre)	110/31	DSC: 0.71, TPR: 0.66, PPV: 0.83, HD95: 2.82
[28]	T1, FLAIR / *1.5–3T*	3D transformer model, *S*	LISA - healthy older individuals / MICCAI 2017 WMH Challenge* (multi-centre)	240/20	DSC: 0.84, TPR: 0.40, AVD: 0.56, F1: 0.53, HD: 14.02, FPR: 0.00013
[49]	T1, T2, FLAIR / *1.5–3T*	Slime Mold Algorithm and Harris Hawk's Optimization algorithm to train DCNN, *S*	In house dataset (range of neurological disorders) and MICCAI 2017 WMH Challenge* (multi-centre)	276/74/550	DSC: 0.87, TPR: 0.89, HD95: 6.21, AVD: 0.21, PPV: 0.90, F1: 0.89
[75]	FLAIR / *3T*	Instance loss functions, *S*	ADNI-GO* / MICCAI 2017 WMH Challenge* (both multi-centre)	20/60 – 5-fold CV for training/ validation	DSC: 0.61, TPR: 0.43, PPV: 0.47, F1: 0.46, FNR: 0.57, FDR: 0.39
[12]	T1-MPRAGE, T2-SPACE, FLAIR / *1.5T*	Hyperspectral algorithm - iterative CEM and kernal CEM, *S*	Patients with AD	10 total images - 300 training samples	DSC: 0.87–0.93
[18]	T1, FLAIR / *1.5–3T*	CNN, *S*	Longitudinal study of patients with CADASIL	Preliminary model: 22/9/59 Final model: 25/5/20	DSC: 0.83, TPR: 0.71, AVD: 0.26, F1: 0.49, HD: 6.67

** Code publicly available, * Data available.

CEM: constrained energy minimization, CLoss: combined loss function, CNN: convolutional neural network, CRF: conditional random fields, CV: cross validation, EM: expectation-maximization, EVT: extreme value theory, GLCM: gray level co-occurrence matrix, GMM: Gaussian mixture model, HeMIS: hetero-modal image segmentation, k-NN: k nearest neighbours, LSLoss: level set loss function, MRF: Markov Random Field, NNR: nearest neighbour refinement, SVM: support vector machine.

*RB*: rule-based, S: supervised, *SS*: semi-supervised, *US*: unsupervised.

CSVD: cerebral small vessel disease, ICH: intracerebral hemorrhage, T2DM: type 2 diabetes mellitus, TIA: transient ischemic attack, WM: white matter, WMH: white matter hyperintensity.

DSC: dice sensitivity coefficient, DER: detection error rate, FPavg: average number of false positives per scan/subject, FNR: false negative ratio, FPR: false positive ratio, OER: outline error rate, PPV: positive predictive value (also referred to as precision), TPR: true positive ratio (also referred to as recall or sensitivity).

ABVIB: Aging Brain: Vasculature, Ischemia, and Behavior study, ADC: Alzheimer’s Disease Center, ADNI: Alzheimer’s Disease Neuroimaging Initiative, AGES: Age, Gene/Environment Susceptibility, AIBL: Australian Imaging Biomarkers and Lifestyle, BIOCARD: Biomarkers of Cognitive Decline Among Normal Individuals: the BIOCARD cohort, CAIN: Canadian Atherosclerosis Imaging Network, CAMERA: community study of Cardio- and cerebrovascular Accident Monitoring, Epidemiology, and caRe quAlity system, CNSR: Chinese National Stroke Registry, ENVIS-ion: aspirin for the prevention of cognitive decline in the Elderly: rationale and design of a Neuro-Vascular Imaging Study, HKU-SVD: University of Hong Kong small vessel disease cohort, ISLES: Ischemic Stroke Lesion Segmentation, JPSC-AD: Japan Prospective Studies Collaboration for Aging and Dementia, KLOSCAD: Korean Longitudinal Study on Cognitive Aging and Dementia, KHP-DCR: Kings Health Partners-Dementia Case Register, LIPA: Language Impairment in Progressive Aphasia, LISA: Live active Successful Aging, MEMORA: Predictive Factors of the Autonomy Level Change Related to Memory Disorders, MITNEC: Medical Imaging Trial Network of Canada, MRI-GENIE: MRI-GENetics Interface Exploration, NACC: National Alzheimer’s Coordinating Center, NIFD: FrontoTemporal Lobar Degeneration Neuroimaging Initiative, OATS: Older Australian Twins Study, ONDRI: Ontario Neurodegenerative Disease Research Initiative, OPTIMA: Oxford Project to Investigate Memory and Ageing, OXVASC: Oxford Vascular Study, PREVENT-AD: PRe-symptomatic EValuation of Experimental or Novel Treatments for Alzheimer’s Disease, RUN DMC: Radboud University Nijmegen Diffusion tensor and Magnetic resonance imaging Cohort, SAMS: Sydney Memory and Ageing Study, UDES: Utrecht Diabetic Encephalopathy Study, VBH: Vascular Brain Health study, WHICAP: Washington Heights Inwood Columbia Aging Project.

Other external data sources for studies included the 2015 MICCAI Ischemic Stroke Lesion Segmentation (ISLES) challenge [63], which provided two public datasets for two different challenges. The first sub-acute ischemic stroke (SISS) dataset consisted of 28 training cases from a single centre, and 36 cases from two centres. The second acute ischemic stroke (SPES) dataset consisted of 30 training cases and 20 testing cases from the same centre. Additional challenge datasets include the MR Brain Segmentation Challenge 2013 (MRBrainS13) [66] which provided 20 T1, T1-IR, and FLAIR images for 20 cases, with 5 fully annotated (white matter, grey matter, and CSF) for training, and the revised MRBrainS18 which provides 7 fully annotated (10 labels including WMH) T1, T1-IR, and FLAIR images for training and a total of 30 scans from subjects with diabetes, dementia, AD, and matched controls with increased cardiovascular risk. Another external data source used by two of the included studies [43,75] was a publicly available dataset of 20 participants from ADNI scanned 3 times 12 months apart.

T1 and FLAIR were used as model inputs for 35/59 studies while FLAIR alone was used in 14 studies, and the remaining 10 included additional sequences in combination with FLAIR. Most studies utilized supervised machine learning methods (*n* = 45), while one used a semi-supervised method (combined labeled and unlabeled training data), one used a hybrid approach combining unsupervised and supervised techniques, three combined supervised and rule-based methods, three combined unsupervised and rule-based methods, two relied on rule-based algorithms, and four used unsupervised approaches. The rule-based and unsupervised methods used various algorithms to classify WMH from normal white matter using histographic information on intensity and texture. The supervised methods included random forests, regression, k-nearest neighbor (k-NN), support vector machines (SVM), and 2D, 2.5D and 3D convolutional neural networks (CNNs) with most employing architectures based on U-Net.

A variety of methods were used to determine accuracy (Table 3). The most commonly used metric was the DSC, and many additionally reported metrics as outlined in the MICCAI 2017 WMH Challenge including sensitivity (TPR), Hausdorff distance (modified, 95th percentile - HD95), average volume distance (AVD), and F1 score. Of those that reported DSC, the mean was 0.74 and the range was 0.46 to 0.95. The winner of the 2017 MICCAI WMH segmentation challenge, sysu_media, achieved a DSC of 0.80, TPR of 84 %, HD95 of 6.3 mm, AVD of 22 %, and F1-score of 0.76 [53]. Several methods later reported similar or improved performance to sysu_media when trained on the same datasets [55,56,58,105].

Some studies compared their methods to other publicly available segmentation tools for WMH. The most common comparators for this purpose were the two Lesion Segmentation Tool (LST) algorithms–lesion growth algorithm (LGA) and lesion prediction algorithm (LPA)– and the Brain Intensity AbNormality Classification Algorithm (BIANCA) used in the UK Biobank dataset.

Furthermore, four studies compared different machine learning methods within the same dataset in the same study. Ithapu et al. [42] found that random forest performed better than SVM, with both showing higher performance to LGA. Dadar et al. [15] compared 10 different classification techniques for segmentation of WMH in three different neurodegenerative cohorts and found that random forest had the highest performance. Ghafoorian et al. [30] compared configurations of 2D CNN architectures and found that CNNs that integrate spatial location features significantly outperform those that do not, and that the multi-scale late fusion configurations had the highest performance. Duarte et al. [23] compared four feature extraction architectures (VGG16, VGG19, ResNet152, EfficientNetB0) within U-Net variations of three different dimensions (2D, 2.5D, 3D), concluding that the 2.5D implementation with VGG16 or VGG19 was most suitable for WMH segmentation across U-Net, LinkNet, and Feature-Pyramid Network (FPN) variants.

### 3.4 Cerebral microbleed detection

A total of 23 studies met inclusion criteria and described novel methods for the detection of CMB, with details provided in Table 4. In the context of CMB, segmentation refers to lesion count and location (detection) as opposed to volumetric segmentation, as the signal on T2* is poorly related to lesion volume due to the blooming effect. A subset of 20 susceptibility weighted imaging (SWI) images with ground truth segmentations for CMB from Dou et al. [21] are publicly available and were used by seven of the included studies. High and low resolution datasets from [2] were also previously publicly available and used by two subsequent studies but are now restricted.

**Table 4:** Studies describing novel methods for detecting cerebral microbleeds.

Ref	Seq / *Field strength*	Architecture (detection / classification), *type*	Dataset (training/ testing)	Total scans (training/ validation/testing)	Performance metrics
[21] **	SWI / *3T*	FCN / 3D-CNN, *S*	Stroke or normal aging *	230/40/50	TPR: 0.93, PPV: 0.44, FP_avg_: 2.74
[111]	SWI / *3T*	SNP / SLFN, *S*	CADASIL or healthy controls	20 total - 8-fold/1-fold/ 1-fold	TPR: 0.93, specificity: 0.93, accuracy: 0.93
[5]	SWI / *3T*	Statistical thresholding and hole filling / QDA, *S*	Stroke or normal aging [21]	14/6	TPR: 0.94, FP_avg_: 56
[60]	SWI, QSM / *1.5–3T*	3D-FRST / 3D-CNN, *RB+S*	Hemodialysis, TBI, stroke or healthy controls (multi-centre)	154/25/41	TPR: 0.96, PPV: 0.71, FP_avg_: 1.6
[61]	SWI / *3T*	VGG / ELM-GBA, *S*	CADASIL or healthy controls	20 total - 7-fold/3-fold	TPR: 0.93, specificity: 0.87, accuracy: 0.90
[2] **	SWI, QSM / *3T*	2D-YOLO / 3D-CNN, *S*	Two in-plane resolutions of MRs with CMBs	107 low res, 72 high res - 5-fold CV	TPR: 0.92–0.94, PPV: 0.62–0.67, FP_avg_: 1.42–1.89, F1: 0.75–0.78
[22]	T2* / *1.5–3T*	U-Net, *S*	CNSR-III - Ischemic stroke or TIA (multi-centre)	359/30	DSC: 0.50, F1: 0.71
[1]	SWI / *3T*	K-mean clustering and GLCM / CNN, *US+RB+S*	Stroke or normal aging [21]	20 total split 80/20 %	TPR: 0.99, PPV: 0.97, FPavg: 1.5, specificity: 0.98, accuracy: 0.99
[14]	T1, SWI or T2* / *3T*	Canny edge detection and circular Hough transform, *RB+US*	WHICAP - Aging cohort with and without CMBs (longitudinal)	78	TPR: 0.92–0.95, PPV: 0.07–0.11, FP_avg_: 9.7–17.1
[54]	SWAN / *3T*	FE layer in SSD-512 algorithm, *S*	Patients with CMBs	33/8/17	TPR: 0.9, PPV: 0.80, FP_avg_: 0.23
[62]	SWI / *3T*	2D-CNN / ensemble of SNN, RVFL and ELM, *S*	CADASIL or healthy controls	20 total – 5-fold CV	TPR: 0.98, PPV: 0.99, specificity: 0.99, accuracy: 0.99, F1: 0.99
[70]	T2* / *3T*	YOLO V2 with CSF filtering, *S+RB*	Anonymized	140/46	TPR: 0.67, PPV: 0.80, FP_avg_: 2.15, F1: 0.73
[76] **	SWI, QSM / *3T*	U-Net with padded convolutions, *S*	MESA - Atrial fibrillation (multi-centre)	17/6/1 - leave- one-out CV	TPR: 0.84, PPV: 59
[29]	SWI / *3T*	ResNet50 / Fast R-CNN, *S*	(Al-Mansi et al., 2020) / [21]	179/20 – 5-fold CV	TPR: 0.93, PPV: 0.90, FP_avg_: 0.24, F1: 0.91
[87]	SWI / *3T*	S3DGCM / 1D-CNN, *RB+S*	[21] and [2] datasets	230/90 and 107/72 – 10-fold CV	TPR: 0.99 and 0.98, FP_avg_: 3.46 and 2.59, specificity: 0.97, accuracy: 0.98, F1: 0.99 and 0.98
[26]	SWI / *1.5–3T*	3D-U-Net, *S*	CSVD / CNSR-III - stroke or TIA (both multi-centre)	1285/330/72	TPR: 0.85, PPV: 0.75, DSC: 0.72, specificity: 0.78–0.84, accuracy: 0.93
[27]	SWI / *3T*	2.5D-CNN, *S*	Stroke or normal aging [21]	14/6	TPR: 0.98, PPV: 0.94, FP_avg_: 1.72
[90] **	T2* or SWI / *3T*	FRST / student- teacher framework, *RB+S*	UK Biobank and OXVASC - stroke or TIA / TICH2 - spontaneous ICH, [21]	44–78/10/24–135	TPR: 0.81–0.90, PPV: 0.62–0.89, FP_avg_: 0.5–3.1
[4]	SWI / *3T*	Thresholding / random forest, *RB+S*	Stroke or normal aging [21]	14/6	TPR: 0.87, FP_avg_: 44, accuracy: 0.97
		Thresholding / naïve Bayes, *RB+S*			TPR: 0.90, FP_avg_: 57.5, accuracy: 0.98
[106]	SWAN, SWI / *3T*	Mask R-CNN, *S*	aSVD, CAA, CADASIL (multi-centre)	336/28	TPR: 0.71, DSC: 0.63
[51] **	SWI / *3T*	EfficientDet (2D-CNN) ensemble, *S*	With or without CMBs (multi-centre)	85/17/25, additional 79 for external testing	TPR: 0.85–0.96, PPV: 0.77–0.80, FP_avg_: 0.55–0.88
[107]	QSM / *3T*	2.5D FRST and CNN / V-Net, *RB+S*	CSVD	280/35/78	TPR: 0.89, PPV: 0.50, FP_avg_: 2.87
[112]	T2 / *1.5–3T*	2D VB-Net, *S*	Lacunar cerebral infarction and controls	294	TPR: 0.77, PPV: 0.76

* Data publicly available for subset of 20 images with ground truth labels, ** Code and/or model publicly available.

CNN: convolutional neural network, CV: cross validation, ELM: extreme learning machine, FE: feature enhancement, FCN: fully convolutional network, FRST: fast radial symmetrical transform, GBA: Gaussian-map bat algorithm, GLCM: gray level co-occurrence matrix, KBPNet: Knowledge-guided Body Plane Network, SLFN: single-hidden layer feed-forward neural-network, SNP: slice neighbourhood processing, SSD: single shot detector, SVM: support vector machine, S3DGCM: Selective 3D Gradient Co-occurrence Matrix, QDA: quadratic discriminant analysis.

*RB*: rule-based, *S*: supervised, *US*: unsupervised.

CAA: cerebral amyloid angiopathy, CADASIL: cerebral autosomal dominant arteriopathy with subcortical infarcts and leukoencephalopathy, CMB: cerebral microbleed, CSVD: cerebral small vessel disease, ICH: intracerebral hemorrhage, TIA: transient ischemic attack.

DSC: dice sensitivity coefficient, FP_avg_: average number of false positives per scan/subject, PPV: positive predictive value (also referred to as precision), TPR: true positive ratio (also referred to as recall or sensitivity).

CNSR: Chinese National Stroke Registry, MESA: Multi-Ethnic Study of Atherosclerosis, OXVASC: Oxford Vascular Study, TICH2: Tranexamic acid for intraCerebral hemorrhage 2 trial, WHICAP: Washington Heights Inwood Columbia Aging Project.

Most studies (14/23) trained and tested their methods using SWI (combined gradient echo (GRE) magnitude and phase) images. Three studies trained and tested their models using only T2* GRE magnitude images [22,70,112], while two others used either GRE magnitude or SWI as inputs to improve model applicability to multicenter studies with a variety of sequences [14,90]. Liu et al. [60] and [2] used both SWI and their complement phase images as two-channel inputs to improve detection performance. Rashid et al. [76] compared inputs of SWI only, SWI and quantitative susceptibility mapping (QSM), SWI and T2, and SWI, QSM and T2, with SWI and QSM giving the highest performance. Only one included study [107] used QSM as the sole input.

Fifteen studies used supervised learning techniques, six studies used hybrid supervised and rule-based approaches, one study used an approach that combined unsupervised, supervised, and rule-based methods, and one study used an unsupervised rule-based approach. The single study that took an unsupervised rule-based approach used canny edge detection and the circular Hough transform, which they referred to as MAGIC [14]. While able to achieve high sensitivity (referred to as TPR) of 92–95 % and an average false positives per subject of <20 for either GRE magnitude or SWI input, precision (also termed positive predictive value [PPV]) was only 7–11 %. The other studies used predominantly supervised networks usually with a two-stage strategy, with the first stage for detection of CMB-like candidates and the second stage for classification and false positive (FP) removal, with hybrid methods using rule-based algorithms for initial candidate selection and/or post-processing.

The most commonly reported metrics to report detection accuracy for CMB were TPR, PPV, and the average number of false positives per scan/subject (FP_avg_). The average TPR was 89.5 % with a range of 66.9 to 99.9 %. The average PPV was 70.6 % with a range of 11 to 98.9 %. FP_avg_ ranged from 0.24 to 56 false positives per subject.

### 3.5 Perivascular space detection and segmentation

A total of six studies met inclusion criteria and described novel methods for the segmentation and detection of PVS with details provided in Table 5. We did not find evidence of publicly available annotated datasets for segmentation algorithm development and testing.

**Table 5:** Studies describing novel methods for detecting and segmenting perivascular spaces.

Ref	Seq / *Field strength*	Task	Architecture, *type*	Dataset (training/ testing)	Total scans (training/ validation/testing)	Performance metrics
[9]	T1, FLAIR, T2/PD / *3T*	Segmentation	Multi-sequence intensity modeling / morphological constraint algorithm, *RB*	Older adults without dementia	14 × 2	Count: *r* = 0.74, p<.001 Volume: *r* = 0.58, p<.01
[83]	T1, FLAIR / *3T*	Quantification	T1 voxel intensity inhomogeneity assessment / morphological constraint algorithm, *RB*	Older adults without dementia, ADNI2	14 and 30	PPV: 0.77–0.87 Subject-wise false alarm frequency: 15–25 % Count: *r* = 0.72, *p* < 0.0001
[24]	T2 / *1.5T*	Quantification	Global pooling U-Net, *WS*	Older adults from the Rotterdam Study	1000/202/1000	TPR: 0.62, FP_avg_: 2.33
[86]	T1, T2-TSE / *7T*	Quantification	k-NN, *S*	UDES2 and PREDICT-MR - with and without cognitive impairment	50 - leave-one-out CV	DSC: 0.61, ICC for count (absolute/consistency): 0.64/0.75
[39] **	T1 / *3T*	Segmentation	nnU-Net, *S*	ADNI3 - Cognitively normal and impaired with high PVS	40/15	DSC: 0.80, TPR: 0.79, PPV: 0.81
[112]	T2 / *1.5–3T*	Quantification	2D VB-Net, *S*	Lacunar cerebral infarction and controls	294	TPR: 0.97, PPV: 0.92

** Code publicly available.

CV: cross validation, k-NN: k nearest neighbours.

*RB*: rule based, *S*: supervised, *US*: unsupervised, *WS*: weakly supervised.

DSC: dice sensitivity coefficient, FP_avg_: average number of false positives per scan/subject, ICC: intra-class correlation coefficient, PPV: positive predictive value (also referred to as precision), *r*: Pearsons’s correlation coefficient, TPR: true positive ratio (also referred to as recall or sensitivity).

ADNI: Alzheimer’s Disease Neuroimaging Initiative, UDES: Utrecht Diabetic Encephalopathy Study.

Two of the included studies evaluated their models on T2 images [24,112]. The other studies used either T1 images [39]; T1, T2/PD, and FLAIR [9]; T1 and FLAIR [83]; or T1 and T2-TSE [86]. Three of the included studies utilized supervised deep learning based approaches [39,86,112], while one used a weakly supervised (optimized with global labels as opposed to local) method [24] and the other two used rule-based methods [9,83]. All of the included studies provided counts of PVS, while two additionally described correlations with volume output [9,39].

Boespflug et al. [9] used multi-sequence intensity modeling and a morphological constraint algorithm accounting for parameters of width, volume, linearity, and location, named MR imaging–based multimodal autoidentification of PVS (mMAPS). Validated against a manual count of PVS in one hemisphere on a single slice, they found good correlations with automated counts (*r* = 0.74, *p* > 0.001) and segmented volume (*r* = 0.58, *p* < 0.01). Schwartz et al. [83] similarly used a morphological constraint algorithm but paired it with a T1 voxel intensity inhomogeneity assessment, resulting in good correlation of automated counts with manual counts (*r* = 0.72, PPV = 0.77–0.87) but using only T1 and FLAIR data, without needing T2/PD. Dubost et al. [24] implemented and compared five machine learning methods and proposed that Global Pooling (GP)-Unet, a weakly supervised method that computes attention maps using a global regression objective, had the highest accuracy. When tested on a brain dataset of 2202 scans collected from the Rotterdam Study [41], an average TPR of 62 % (based on correctly identified lesion counts) was achieved with 2.33 false positives per scan. However, post-hoc review by the human expert raters indicated that many of the so-called “false positives” were probably PVS that were missed by the human raters, highlighting the challenge of training models when the ground truth is subject to intra-rater unreliability. A k-NN based method developed by Spijkerman et al. [86] outputs count, length, and tortuosity of PVS in the centrum semiovale from 3D T2-TSE scans at 7.0 T They achieved a DSC of 0.62 against manually annotated PVS voxels on a single example slice, but the method is of uncertain applicability to 2D T2 obtained at lower field strength, on which PVS are expected to exhibit lower contrast. Huang et al. [39] developed a multi-center PVS segmentation network (mcPVS-Net) which segmented PVS from T1 images across multiple ADNI3 sites. mcPVS-Net achieved a DSC of 0.80, TPR of 79 %, and PPV of 81 % in the test dataset based on correctly identified voxels, compared to a previously developed openly available method from Boutinaud et al. [10] (excluded from the current analysis because it was tested on only young healthy subjects) which achieved a mean DSC of 0.51, TPR of 52 %, and PPV of 54 % in the same dataset. A 2D VB-Net was validated by Zhang et al. [112] on PVS alongside other CSVD lesions, and reported high performance (TPR of 97 %, PPV of 92 % for PVS quantification in a study population of participants with lacunar cerebral infarction and controls.

### 3.6 Lacune and recent small subcortical infarct detection and segmentation

A total of five studies met inclusion criteria for the segmentation of lacunes and RSSI, with details provided in Table 6. RSSI appear as hyperintense lesions on diffusion weighted imaging (DWI) and are found in areas supplied by small penetrating arteries. Only acute and subacute infarcts are hyperintense on DWI. Lacunes appear on FLAIR images as central CSF-like hypointensities, usually surrounding a hyperintense rim. No studies provided evidence of a publicly available annotated dataset.

**Table 6:** Studies describing novel methods for detecting, segmenting, and classifying lacunes and recent small subcortical infarcts.

Ref	Seq / *Field strength*	Task, *lesion type*	Architecture, *type*	Dataset (training/ testing)	Total scans (training/ validation/testing)	Resolution for test set / Performance metrics
[98]	T1, T2 / *1.5T*	Detection / false positive reduction, *lacunes*	Top hat transformation and multiple-phase binarization / SVM, *RB+S*	Participants with suspected lacunar infarcts	132 – two-fold CV	TPR: 0.968, FP_avg_: 0.47/slice
[31]	FLAIR, MPRAGE / *1.5T*	Detection / false positive reduction, *lacunes*	FCN / 3D CNN, *S*	RUN DMC and FUTURE - SVD, stroke, TIA	868/96/111	TPR: 0.974, FP_avg_: 0.13/slice
[22]	FLAIR, T1 / *1.5–3T*	Segmentation, *lacunes*	U-Net, *S*	CNSR-III - Ischemic stroke or TIA (multi-centre)	824/30	DSC: 0.496, F1: 0.683
	DWI / *1.5–3T*	Segmentation, *recent small subcortical infarcts*	U-Net, *S*	CNSR-III - Ischemic stroke or TIA (multi-centre)	1010/30	DSC: 0.728, F1: 0.859
[3] **	FLAIR, MPRAGE / *3T*	Classification, *lacunes*	3D ResNet, *S*	Participants with lacunar infarcts	288 – 5-fold CV	TPR: 0.96, PPV: 0.91, FP_avg_: 1.32, specificity: 0.91, accuracy: 0.94
[112]	T1 / *1.5–3T*	Detection, *lacunes*	2D VB-Net, *S*	Lacunar cerebral infarction and controls	294	TPR: 0.33, PPV: 0.43
	DWI / *1.5–3T*	Segmentation, *recent small subcortical infarcts*	2D VB-Net, *S*	Lacunar cerebral infarction and controls	294	DSC: 1.00, TPR: 1.00, PPV: 1.00

** Code publicly available.

CNN: convolutional neural network, CV: cross validation, FCN: fully convolutional network, SVM: support vector machine.

*RB*: rule-based, *S*: supervised.

SVD: small vessel disease, TIA: transient ischemic attack.

DSC: dice sensitivity coefficient, FP_avg_: average number of false positives per scan/subject, PPV: positive predictive value (also referred to as precision), TPR: true positive ratio (also referred to as recall or sensitivity).

FUTURE: Follow-Up of transient ischemic attack and stroke patients and Unelucidated Risk factor Evaluation study, RUN DMC: Radboud University Nijmegen Diffusion tensor and Magnetic resonance imaging Cohor.

Uchiyama et al. [98] combined rule-based algorithms and supervised SVM for detecting candidate lacunar lesions, for subsequent review by a human reader. The study improved upon their previously published methods to reduce FPs by applying eigenspace template matching and managed to eliminate 34.1 % of FPs compared to the previous method, achieving a TPR of 96.8 % and 0.47 FPs per slice. The remaining studies used supervised approaches. Ghafoorian et al. [31] proposed a novel two-stage CNN-based method trained and tested on a large dataset consisting of 1075 cases from two different studies. A fully convolutional network was utilized for the first stage to reduce the number of candidate lesions, and then a more computationally extensive 3D CNN was used to reduce false positives to 0.13 per slice, reporting a TPR of 97.4 %. Al-Masni et al. [3] developed a semi-automated 3D ResNet classifier that differentiates between true lacunes and lacune mimics by exploiting contextual information using multi-scale anatomical location data, achieving a TPR of 96 %, PPV of 91 %, specificity of 91 %, accuracy of 94 %, and 1.32 FPs per subject. However, the method requires an input of potential lacune candidates from the user.

Two of the five studies also described simultaneous segmentation of other CSVD lesions alongside lacunes [22,112]. Duan et al. [22] combined four segmenter networks based on U-Net architecture, using information from T1, T2*, FLAIR, and DWI to segment WMH, CMB, lacunes, and RSSI. The generated WMH mask is subtracted from both the lacune and RSSI masks, and the lacune mask is further subtracted from the RSSI mask to reduce FPs. A DSC of 0.496 was reported for lacunes, and a DSC of 0.728 for RSSIs. The 2D VB-Net presented in Zhang et al. [112] was validated for segmentation of WMH, CMB, lacunes, RSSI, and PVS, but rather treats each lesion as a separate task with no reported subtraction of specific sequences to improve accuracy. A TPR of 33 % and PPV of 43 % was reported for lacune detection, and segmentation of RSSI was reported as perfect (DSC 1.0, TPR and PPV of 100 %).

## 4.0 Discussion

Visually identified CSVD lesions, such as WMH, CMB, PVS, and lacunes, are strong predictors of risk for stroke and dementia, independent of vascular risk factors [45]. Lesions are often classified as present or absent, counted, or graded by severity into a limited number of categories (*e.g.*, Fazekas scale, Enlarged PVS rating scale). There are multiple downsides to visual review, including lack of granularity, the time needed for review, and imprecision introduced by imperfect inter-rater and intra-rater agreements. These limitations can be addressed by machine learning, which could improve efficiency (depending on the amount of labour needed for preprocessing and managing the analysis pipeline) and precision in quantifying lesion volumes, without suffering from human rater inconsistency (although reliability across scanner types and computing environments still needs to be considered). Precise quantification enabled by machine learning should provide greater statistical power for identifying risk factors and for detecting change over time, which is needed to plan clinical trials of interventions to prevent progression of CSVD. However, to employ quantitative machine learning widely, segmentation methods are needed that are accurate, robust across different scanners, and available for download and use by multiple research groups.

In this systematic review, we identified 89 novel tools (59 for WMH, 23 for CMB, six for PVS, five for lacunes, and two for RSSI) that are presented within the last 10 years and validated on populations diagnosed with or at risk for CSVD (older adults or patients with cognitive disorders). Of these tools, 30 (23 for WMH, five for CMB, one for PVS, and one for lacunes) were included in which authors made them downloadable for use, including one commercial tool [95] and one that depended on a commercial tool for preprocessing [71]. The extent of their ease of use varied – most provided code for Python implementation, while 10 (six for WMH, three for CMB, and one for PVS) provided pretrained models [15,21,39,51,90,92,93,97,99,115], seven provided Docker images for WMH segmentation [15,43,53,69,73,92,105], two are available within FSL [34,91], and six provided a fully implementable toolbox, software, or pipeline for WMH segmentation [15,17,42,44,55,69]. Docker images are packages with everything needed to run the software which can be run consistently across different systems and are becoming a widely adopted standard for sharing and running code due to their reproducibility. Best practice for sharing includes both a Docker image and source code to enable customization. Across all eligible studies, metrics for testing varied, with the DSC being most commonly measured for WMH methods, and sensitivity being most common for other lesion types. Generally, we found that most tools reported good to excellent accuracy against a reference standard of manual or manually corrected segmentations or lesion counts. Similarly, we found the quality of the included studies to be good to excellent, as measured using METRICS.

We found far more studies of WMH than other lesion types, suggesting that segmenting WMH may be the most tractable problem within current machine learning methods. Also, because WMH are more common than other CSVD lesion types, except possibly PVS, more WMH data are available including several downloadable annotated datasets. The mean DSC across all studies was 0.74, which can be considered good to excellent [116], particularly considering that many individual WMH lesions are small with fuzzy borders and therefore are more challenging to segment than, for example, tumours or large territory acute ischemic stroke.

Our review focused on studies that developed and validated new segmentation methods, not on comparative studies. However, some studies comparing segmentation methods for WMH in external datasets are beginning to emerge. LST and BIANCA appear to be the most common comparators. LST implements two algorithms: a lesion growth algorithm (LGA) which is an unsupervised intensity-based method that requires a T1 image in addition to FLAIR, and a lesion prediction algorithm (LPA) based on a regression classifier previously trained on a multiple sclerosis (MS) dataset. While LST was developed in patients with MS, and thus did not meet eligibility criteria for our systematic review, it has been reported to produce accurate segmentation in patients with CSVD as well [78,94], even though vascular lesions exhibit a different load, morphology, and distribution, and generally have less well-defined borders than MS lesions [7]. BIANCA [34] is based on k-NN, and the method was optimized on two different datasets: a predominantly neurodegenerative dataset, and a predominantly vascular dataset (results shown for vascular cohort in Table 3). Several options are available in BIANCA including the flexibility to apply multiple MRI modalities, and the application of spatial weighting and/or a “patch” option for local intensity averaging. BIANCA is available within FSL but requires a manually labeled training dataset when applying data acquired from a new scanner or protocol. BIANCA has also been tested externally and found to produce valid results [37,94], including optimization and validation for segmenting extensive WMH [57]. A clinic-based study found that LST-LPA and BIANCA had higher DSC than LST-LGA and SAMSEG (available as part of FreeSurfer, developed for segmentation of MS lesions [11]) [94]. A study in a convenience sample of older persons found that BIANCA had the highest DSC when using 3D FLAIR but similar DSC to UBO Detector on 2D FLAIR [37]. A population-based study suggested that LST-LPA and LST-LGA are easier to use than BIANCA and had higher DSC in older persons with cardiovascular risk factors [67]. A study using the ADNI database found that the three top-ranked methods were LPA, SLS (MS Lesion Segmentation toolbox [80]), and BIANCA, which had essentially the same performance [100]. Finally, a multicenter study on subjects with vascular cognitive impairment found that kNN-TTP, LST-LGA and LST-LPA had the highest DSC compared to Cascade and Lesion-TOADS [36]. Thus, while comparator studies to date do not support a single clearly superior method, and are limited in the number of methods tested, they identify that there are several valid options for users to choose from. Future comparative studies should focus on current state-of-the-art methods (*e.g.,* TrUE-Net, HyperMapp3r) as benchmarks in addition to comparisons with legacy tools such as LST and BIANCA.

Other CSVD lesion types have, so far, received less attention than WMH. The more recent Where is VALDO? (VAscular Lesions Detection and segmentiOn) challenge [89] at MICCAI 2021 represents an effort to draw more attention to other lesion types, and had 12 teams participate in the development of automated methods for the segmentation of PVS (4 teams), CMB (9 teams), and lacunes (6 teams), with all but one team making use of deep learning. 11/12 participating teams made their docker containers publicly available (https://hub.docker.com/r/whereisvaldo/challenge2021/tags); however, none of these methods were identified in publications in the current review. After WMH, the second largest group of studies were for CMB, of which 5 made their algorithm or code available for use by other research groups. For most of these studies, the purpose was to identify possible CMB with high sensitivity, intentionally sacrificing precision. For example, one method identified approximately 9 false positives for every true positive. Thus, in many cases, these methods require visual review for confirmation, with editing to remove false positives. Even so, these methods could substantially reduce the burden of visual rating by allowing the human rater to focus on a limited subset of possible CMB, without having to review every susceptibility artifact in the image.

Similar to WMH, PVS are usually visible, to some extent, on MRI in older populations, and a higher grade on visual rating scales is associated with CSVD [50]. However, we found much fewer machine learning methods for PVS measurement that were validated on a representative dataset. Notably, 42/48 studies were excluded at full text review, with 21 presenting novel methods. Of these, six studies validated their segmentation on a cohort of young healthy adults (age 25–37). Models developed and validated on young healthy adult datasets are likely to perform inadequately in aging and diseased cohorts due to their potential interference with WMH and other brain lesions [39]. In addition, the presence of PVS on T2 images in young healthy adults suggests that these features may not indicate pathological changes [8]. Overall, the reduced focus on PVS segmentation is probably due to increased difficulty in accurately identifying PVS compared to lesions like WMH, as suggested by lower DSC and TPR scores for the PVS methods. Of the studies presented, only one study provided code for download. Of the four methods presented in the Where is VALDO? challenge, three utilized U-Net based models (nnUNet, MaskRCNN, UNet) and the top performing team used a random forest-based method, indicating that PVS may be easier to characterize in terms of signal and shape signature. Within the challenge, teams were required to both detect and segment PVS; however, only two out of six of the included studies found in this review [9,39] provided correlations with PVS volume in addition to PVS count. It should be noted that PVS volume better captures disease severity and is more sensitive to change over time and has thus been recommended as an important metric in addition to PVS count in clinical studies [74]. We recommend that future studies should report descriptive statistics on their PVS outputs, including volumes as well as PVS counts.

So far, lacunes and RSSI have received much less attention than segmentation for acute infarcts in acute ischemic stroke [103]. We found only five studies, of which one provided source code for download: a semi-automated classifier that differentiates between true lacunes and lacune mimics [3]. Interestingly, two of the methods were also validated on other CSVD lesion types [22,112], and one performed the segmentations simultaneously [22], an approach that might improve discrimination between lesion types and be convenient and efficient for users.

Some limitations should be kept in mind when interpreting the results of this systematic review. The review was limited to the English language and publications presented in the 10 years prior to September 2024. Due to the rapid development of the field, there are likely methods available that are not presented in this review. Because we focused on initial derivation and validation, studies comparing different established methods may not have met our selection criteria; however, larger scale comparative studies were summarized previously in this discussion. To ensure that studies were applicable to CSVD in older persons, we excluded studies of WMH and PVS in other disease states (*e.g.*, MS) and in young adults; however, some of the tools derived in other diseases or age ranges, such as the LST tool for WMH, have subsequently been applied and validated in older adults too.

We also identified limitations of the current literature and can offer some perspectives on a future research agenda. Generally speaking, all of the methods would benefit from further external validation, ideally including studies by research groups that are independent of the original developers. According to the HARmoNizing Brain Imaging MEthodS for VaScular Contributions to Neurodegeneration (HARNESS) framework for neuroimaging biomarker development [85], most of the tools exhibit published data on proof of concept (*i.e.*, that the tool measures what it purports to), repeatability (precision under the same operating conditions), proof of principle (ability to separate CSVD cases or burden), and, in some cases, reproducibility (precision on different scanners). However, there are aspects of validation that are less well developed such as cost (including time saved from avoiding manual visual review), reproducibility across different computing environments, and proof of effectiveness, defined by HARNESS as the ability to measure endpoints across larger groups of patients in multi-center studies. The ultimate validation comes from incorporating tools as endpoints in practice-changing clinical trials and using them routinely for diagnosis in radiology departments.

Another limitation is that segmentation methods for CSVD lesion types other than WMH—CMB, PVS, and lacunes—are still at an early stage of development; however, early studies for all lesion types suggest that they may be amenable to segmentation using machine learning. The production of publicly available annotated datasets, particularly for PVS and lacunes, and those that include multi-centre and variable loads representing the broader population at risk, would accelerate development of novel methods. Deriving methods that can simultaneously segment multiple lesion types accurately might be possible and could improve the distinction of lesion types that share some characteristics (*e.g.,* distinguishing small WMH from PVS from lacunes), enhancing accuracy and efficiency. Finally, studies are needed on how these methods can be integrated into routine radiological practice and how they would affect clinical diagnosis and treatment.

In conclusion, this review found good evidence that valid WMH segmentation methods are available for research use, but fewer data for other CSVD lesion types. CMB segmentation methods are currently suitable for identifying candidate lesions for visual review and confirmation, but more work is needed to develop fully automated methods that don’t require human oversight. Tools for PVS and lacunes are at an earlier stage of development but are emerging.

## Funding sources

This work was supported by the Canadian Institutes of Health Research (grant RT0 179993).

## CRediT authorship contribution statement

**Jolene Phelps:** Writing – original draft, Methodology, Investigation, Formal analysis, Data curation, Conceptualization. **Manpreet Singh:** Writing – review & editing, Methodology, Investigation, Formal analysis. **Cheryl R. McCreary:** Writing – review & editing, Methodology, Investigation. **Caroline Dallaire-Théroux:** Writing – review & editing, Methodology, Investigation. **Ryan G. Stein:** Writing – review & editing, Methodology, Investigation. **Zacharie Potvin-Jutras:** Writing – review & editing, Methodology, Investigation. **Dylan X. Guan:** Writing – review & editing, Methodology, Investigation. **Jeng-liang D. Wu:** Writing – review & editing, Methodology, Investigation. **Amelie Metz:** Writing – review & editing, Investigation, Funding acquisition. **Eric E. Smith:** Writing – review & editing, Writing – original draft, Supervision, Resources, Project administration, Methodology, Investigation, Conceptualization.

## Declaration of competing interest

The authors report no competing interests.

## Appendix Supplementary materials

Image, application 1

Image, application 2
