# Supplementary material for: Cerebral small vessel disease lesion segmentation methods: A systematic review
Source: Cereb Circ Cogn Behav. 2025 Sep 19;9:100396. doi: 10.1016/j.cccb.2025.100396 (PMC12513155; doi:10.1016/j.cccb.2025.100396)
Supplement: Supplementary file 1 [file mmc1.docx]

**APPENDIX A. Search Strategy**

Web of Science:

ALL=("white matter hyperintens*" OR "wmh" OR "leukoaraiosis" OR "white matter lesion*" OR "wml" OR "white matter chang*" OR "leukoencephalopath*" OR "white matter disease*" OR "white matter damage*" OR "subcortical infarct*" OR "rssi" OR "lacunar infarct*" OR "perivascular space*" OR "pvs" OR "Virchow Robin" OR "Virchow-Robin" OR "état crible" OR "superficial siderosis" OR "microinfarct*" OR "microischemia" OR "microvascular ischemia" OR "microvascular infarct*" OR "cerebral microbleed*" OR "cmb" OR "microbleed*" OR "microhemorrhage*" OR "microhaemorrhage*" OR "dot-like hemosiderin" OR "dot-like haemosiderin" OR "lacune*" OR "silent brain infarct*" OR "lacunar lesion*")

AND ALL=(“cerebral small vessel disease” OR “dementia, vascular” OR “stroke*” OR “leukoaraiosis” OR “brain*”)

AND ALL=("detection" OR "segmentation" OR “machine learning” OR “deep learning” OR “parcellation” OR “artificial neural network” OR “clustering” OR “classification”)

AND ALL=(“MRI” OR “magnetic resonance imag*” OR “neuroimag*”)

AND DOP=(2013-12-20/2023-12-20)

AND LA=(English)

AND DT=(Article)

MEDLINE/PubMED:

("white matter hyperintens*" OR "wmh" OR "leukoaraiosis" OR "white matter lesion*" OR "wml" OR "white matter chang*" OR "leukoencephalopath*" OR "white matter disease*" OR "white matter damage*" OR "subcortical infarct*" OR "rssi" OR "lacunar infarct*" OR "perivascular space*" OR "pvs" OR "Virchow Robin" OR "Virchow-Robin" OR "état crible" OR "superficial siderosis" OR "microinfarct*" OR "microischemia" OR "microvascular ischemia" OR "microvascular infarct*" OR "cerebral microbleed*" OR "cmb" OR "microbleed*" OR "microhemorrhage*" OR "microhaemorrhage*" OR "dot-like hemosiderin" OR "dot-like haemosiderin" OR "lacune*" OR "silent brain infarct*" OR "lacunar lesion*")

AND (“cerebral small vessel disease” OR “dementia, vascular” OR “stroke*” OR “leukoaraiosis” OR “brain*”)

AND ("detection" OR "segmentation" OR “machine learning” OR “deep learning” OR “parcellation” OR “artificial neural network” OR “clustering” OR “classification”)

AND (“MRI” OR “magnetic resonance imag*” OR “neuroimag*”)

AND (("2013/12/20"[Date - Publication] : "2023/12/20"[Date - Publication]))

AND (English[Language])

NOT (review[Publication type])

AND (humans[Filter])
